# Supplementary material for: Angiopoietin-like 2 is essential to aortic valve development in mice
Source: Commun Biol. 2022 Nov 21;5:1277. doi: 10.1038/s42003-022-04243-6 (PMC9681843; doi:10.1038/s42003-022-04243-6)
Supplement: Supplementary file 2 — Supplementary Information [file 42003_2022_4243_MOESM2_ESM.pdf]

Supplementary Material for Labbé et *al.*

Table S1: Echocardiographic data of *Angptl2*-KD mice and their age-matched WT littermates at 2 months and 3.5 months.

Table S2: Echocardiographic data of *Angptl2*-KD mice and their age-matched WT littermates at 7 months.

Table S3: Primer sequences used in quantitative RT-PCR.

Table S4: Overview of the primary/secondary antibodies.

Supplementary Data 1: Genes significantly regulated in human adult vs. Fetal aortic valves.

Supplementary Data 2: WGCNA turquoise gene module.

Supplementary Data 3: Pathway enrichment of the WGCNA turquoise gene module.

Supplementary Data 4: Source data for main Figures.

Figure S1: Expression profile of *Angptl2* RNA in Eurexpress.

Figure S2: ANGPTL2 expression in sub-cultured mouse VICs.

Figure S3: Specificity of ANGPTL2 staining of E14.5 and 2 month-old mice AoV.

Figure S4: Evaluation of the mitral valve function by echo in *Angptl2*-KD mice.

Figure S5: AVS is still present in 7-10-month old male *Angptl2*-KD mice.

Figure S6: Correlation between the severity of AVS and cardiac hypertrophy in *Angptl2*-KD mice.

Figure S7: Survival curve of *Angptl2*-KD mice.

Figure S8: Changes in gene expression in AoV leaflets from *Angptl2*-KD mice.

Figure S9: Increased black deposits observed in AoV leaflets from *Angptl2*-KD mice.

Figure S10: PirB and Integrin $\alpha5\beta1$ expression in embryonic and adult mice AoV.

Figure S11: WGCNA analysis identified 17 heart valve genetic modules.

Figure S12: Unedited blots/gels for Figure S2.

Table S1: Echocardiographic data of *Angptl2*-KD mice and their age-matched WT littermates at 2 months and 3.5 months.

Data are means±SEM of 5 mice per group (or means±SEM of *n* mice indicated for each parameter); *:< 0.05 versus age- and sex-matched WT mice; Mann-Whitney U tests were used. LV: left ventricular.

2 MONTHS		Male WT (n=5)	Male KD (n=5)
**LV structure**	LV mass (mg)	102±5.07	129±16.0
	Body weight (g)	26.4±0.887	24.7±1.16
	LV mass/body weight (mg/g)	3.84±0.134	5.15±0.491 p=0.056
	LV anterior wall thickness (mm)	0.70±0.04	0.81±0.04 p=0.095
	LV posterior wall thickness (mm)	0.62±0.03	0.75±0.05 p=0.095
**LV systolic function**	Ejection Fraction (%)	56.9±3.74	54.2±4.42
	Fractional shortening (%)	25.7±2.41	24.1±2.51
	Cardiac output (mL/min)	11.6±0.712	12.3±1.46
	Stroke volume (µl)	32.6±1.21	28.8±2.71
	Septal contractility (cm/s)	2.09±0.138	2.01±0.129
	Lateral contractility (cm/s)	1.76±0.124	1.76±0.0942
	LV end-diastolic volume (µL)	193.4±10.23	194.2±23.04
	LV end-systolic volume (µL)	83.8±9.66	91.6±18.3
**LV diastolic function**	E/A ratio	1.22±0.043 (n=5)	1.46±0.30 (n=2)
	E/E' ratio	31.0±2.56 (n=5)	38.7±5.26 (n=2)
	E velocity (cm/s)	63.7±1.44	90.7±4.18 **
	A velocity (cm/s)	52.3±1.69 (n=5)	63.6±12.2 (n=2)
	E deceleration time (ms)	27.2±3.53	30.2±3.73
	E deceleration rate (m/s^2)	25.3±3.21	31.4±3.01
	Iso volumetric relaxation time (ms)	15.8±1.99	11.5±1.34 p=0.0873

2 MONTHS		Female WT (n=5)	Female KD (n=5)
LV structure	LV mass (mg)	77.6±5.62	83.7±6.19
	Body weight (g)	18.6±0.75	17.6±0.68
	LV mass/body weight (mg/g)	4.15±0.17	4.76±0.32 p=0.095
	LV anterior wall thickness (mm)	0.60±0.03	0.71±0.02 p=0.095
	LV posterior wall thickness (mm)	0.54±0.02	0.58±0.04
LV systolic function	Ejection Fraction (%)	53.4±1.56	66.9±4.66 *
	Fractional shortening (%)	23.4±0.874	32.5±3.38 *
	Cardiac output (mL/min)	11.4±1.09	13.4±1.35
	Stroke volume (μl)	29.0±2.81	29.4±2.48
	Septal contractility (cm/s)	1.99±0.15	1.83±0.09
	Lateral contractility (cm/s)	1.83±0.16	1.80±0.08
	LV end-diastolic volume (μL)	166.2±16.50	144.8±11.63
	LV end-systolic volume (μL)	77.2±7.33	49.4±10.9 p=0.0952
LV diastolic function	E/A ratio	1.88±0.22 (n=5)	1.42±0.056 (n=3)
	E/E' ratio	31.1±1.11 (n=5)	34.9±2.23 (n=3)
	E velocity (cm/s)	69.9±6.26	81.9±2.57 p=0.095
	A velocity (cm/s)	39.2±5.50 (n=5)	58.6±1.06 * (n=3)
	E deceleration time (ms)	27.1±1.50	32.1±1.56 p=0.056
	E deceleration rate (m/s^2)	26.2±2.52	25.9±1.80
	Iso volumetric relaxation time (ms)	10.1±1.16	5.44±1.22 p=0.0556

3.5 MONTHS		Male WT (n=5)	Male KD (n=5)
LV structure	LV mass (mg)	113±7.17	128±14.2
	Body weight (g)	30.8±1.66	24.5±1.94 *
	LV mass/body weight (mg/g)	3.65±0.0620	4.90±0.359 *
	LV anterior wall thickness (mm)	0.76±0.04	0.79±0.04
	LV posterior wall thickness (mm)	0.64±0.03	0.76±0.02 *

**LV systolic function**	Ejection Fraction (%)	51.2±2.81	58.4±4.11
	Fractional shortening (%)	22.3±1.57	26.7±2.52
	Cardiac output (mL/min)	12.7±1.22	11.7±1.06
	Stroke volume (µl)	29.2±2.13	24.0±1.38 p=0.087
	Septal contractility (cm/s)	2.13±0.0301	2.26±0.104
	Lateral contractility (cm/s)	2.03±0.0760	2.20±0.117
	LV end-diastolic volume (µL)	198.2±8.327	201.4±28.58
	LV end-systolic volume (µL)	97.4±8.55	88.0±19.0
**LV diastolic function**	E/A ratio	1.43±0.14 (n=4)	1.25±0.095 (n=3)
	E/E' ratio	30.0±2.21 (n=4)	35.8±5.76 (n=3)
	E velocity (cm/s)	69.3±5.57	94.6±7.96 *
	A velocity (cm/s)	46.2±4.16 (n=4)	66.6±5.43 p=0.0571 (n=3)
	E deceleration time (ms)	27.1±2.11	25.3±1.65
	E deceleration rate (m/s^2)	26.1±1.90	37.5±1.98 **
	Iso volumetric relaxation time (ms)	6.76±1.28	6.31±0.901

Table S2: Echocardiographic data of male *Angptl2*-KD mice and their age-matched WT littermates at 7 months.

Data are means±SEM of n=13 WT mice and n=14 *Angptl2*-KD mice (or means±SEM *of n* mice indicated for each parameter); *:< 0.05 versus age- and sex-matched WT mice determined with t-test or Mann-Whitney U test according to the normality of distribution. LV: left ventricular.

7 MONTHS		WT (n=13)	KD (n=14)	p value
**LV structure**	LV mass (mg)	117.7±5.962	123.7±11.31	0.6509
	Body weight (g)	35.2±1.64	29.1±1.13	**0.0053** **
	LV mass/body weight (mg/g)	3.41±0.203	4.17±0.261	**0.0222** *
	LV anterior wall thickness (mm)	0.82±0.027	0.83±0.041	0.7291
	LV posterior wall thickness (mm)	0.75±0.015	0.75±0.034	0.9333
**LV systolic function**	Heart rate (bpm)	460±10(n=9)	455±15 (n=9)	0.3736
	Ejection Fraction (%)	74.6±1.67	66.4±3.09	**0.0316** *
	Fractional shortening (%)	38.2±1.39	32.7±2.36	**0.0583**
	Cardiac output (mL/min)	11.5±0.41	12.0±0.37	0.4481
	Stroke volume (mL)	0.027±0.001	0.028±0.001	0.6673
	Lateral contractility (cm/s)	2.40±0.09	1.87±0.08	**0.0002** ***
	Septal contractility (cm/s)	2.55±0.09	2.18±0.08	**0.0053** **
	LV end-diastolic volume (µL)	166.2±9.923	185.0±21.97	0.3560
	LV end-systolic volume (µL)	42.5±4.30	68.4±12.7	0.1654
**LV diastolic function**	E/A ratio	1.48±0.04 (n=9)	1.53±0.08 (n=5)	0.4970
	E/E' ratio	26.2±2.23 (n=10)	43.4±4.12 (n=5)	**0.0080** **
	E velocity (cm/s)	84.9±2.38 (n=13)	95.2±5.20 (n=13)	0.0828
	A velocity (cm/s)	56.1±1.30 (n=9)	53.7±3.05 (n=5)	0.3636
	Deceleration time (ms)	33.2±1.83 (n=13)	35.0±2.09 (n=13)	0.5195
	Iso volumetric relaxation time (ms)	7.01±0.66 (n=12)	6.69±0.25 (n=13)	0.7163

**Myocardial performance**	Myocardial performance index (global)	43.2±3.01 (n=13)	54.0±2.02 (n=13)	**0.0067** **
	Myocardial performance index (lateral)	63.0±3.65	71.1±3.11	0.1016
	Myocardial performance index (septal)	58.4±3.77	68.1±3.05	0.0554

Table S3: Primer sequences used in quantitative RT-PCR.

Target gene	GenBank Accession (mRNA sequence)	Forward	Reverse	Expected size of amplicon (bp)	Annealing temperature (°C)
Angptl2	NM_011923	GATCCAGAGTGACCAGAATC	TCTCAGGCTTCACCAGGTAG	170	55
Col1a1	NM_007742	TGGTGCCAAGGGTCTCACTG	AGGGCGACCATCTTGACCAG	91	62
Col3a1	NM_009930	CGGGTGCTCCTGGACAGAAT	GTCCAGAACTGCCAGTGGGT	114	62
Mmp2	NM_008610	AGGGCACCTCCTACAACAGC	CATAGCGGTCTCGGGACAGAA	119	61
Bmp2	NM_007553	CAAACACAAACAGCGGAAGC	GTGCCACGATCCAGTCATTC	98	57
Bmp4	NM_007554	GCCCGGAAGCTAGGTGAGTT	CCATCAGGGACGGAGACCAG	102	61
Notch1	NM_008714	TGGATGACCTAGGCAAGTCG	TTCTGCATGTCCTTGTTGGC	103	58
Hes1	NM_008235	ACACCGGACAAACCAAAGAC	AATGCCGGGAGCTATCTTTC	148	57
Hey1	NM_010423	CACCTGAAAATGCTGCACAC	ATGCTCAGATAACGGGCAAC	122	57
Hey2	NM_013904	ACAAGGATCTGCCAAGTTAG	CAATGCTCATGAAGTCTGTG	131	53
Spp1	NM_001204201	CTGTTTGGCATTGCCTCCTC	GTGCAGGCTGTAAAGCTTCTT	81	59
Alp1	NM_007431	GTTGCCAAGCTGGGAAGAACAC	CCCACCCCGCTATTCCAAAC	121	61
Dcn	NM_001190451	TTCCTACTCGGCTGTGAGTC	AAGTTGAATGGCAGAACGC	100	56
Vcan	NM_001081249	CATCGACGCACATGGGATGC	CATGGCCCACACGATTCACA	109	60
Eln	NM_007925	GTCCCAGGTGGTGTTGGAGT	CCACAGCACCTGTCGAGACT	118	61
Ctnnb1	NM_007614	GCGTGGACAATGGCTACTCAAG	TATTAACTACCACCTGGTCCTC	517	58
Axin2	NM_015732	CCTGTGGAACCTGCTGCCTT	TCATCCTCCCGGATCTGCTG	116	61
NF-KB	NM_008689	CACGGATGACAGAGGCGTGTA	AGGCTCCAGTCTCCGAGTGAA	236	64
Sox9	NM_011448	TTCAGATGCAGTGAGGAGCA	CTTGCAGAGGCATGTGTTGT	124	57
TGF-β1	NM_011577	GCTAATGGTGGACCGCAACAAC	CACTGCTTCCCGAATGTCTGAC	100	63
Nppa	NM_008725	GGTAGGATTGACAGGATTGGAG	GACACACCACAAGGGCTTAG	138	58
Nppb	NM_008726	AGGCGAGACAAGGGAGAACA	GGAGATCCATGCCGCAGA	63	59
Myh7	NM_080728	CGGACCTTGGAAGACCAGAT	GACAGCTCCCCATTCTCTGT	116	59
Cyclophilin A	NM_008907	CCGATGACGAGCCCTTGG	GCCGCCAGTGCCATTATG	191	58

Table S4: Overview of the primary/secondary antibodies.

**Antibody**	**Species**	**Dilution**	**Company (catalogue number)**
Angptl2	Goat	1:50	R&D Systems (AF1444)
CD31	Rabbit	1:100	Abcam (ab28364)
CD31	Rat	1:100	R&D Systems (AF3628)
p21	Rabbit	1:100	Abcam (ab188224)
Ki67	Rabbit	1:200	Abcam (ab15580)
Vimentin	Rabbit	1:200	Vector Laboratories (VP-V683)
α-SMA	Mouse	1:100	Sigma-Aldrich (A5228)
Activated-Notch1	Rabbit	1:100	Abcam (ab8925)
Notch1	Rabbit	1:100	Abcam (ab52627)
Notch1	Rabbit	1:100	Cell Signaling (3608S)
Integrin $\alpha 5\beta 1$	Rat	1:100	Sigma-Aldrich (MAB2514)
PirB	Rabbit	1:100	Abcam (ab284407)
Alexa fluor-647	Rabbit	1:500	ThermoFisher (A31573)
Alexa fluor-555	Mouse	1:500	ThermoFisher (A31570)
Alexa fluor-555	Goat	1:500	ThermoFisher (A21432)
Alexa fluor-488	Rat	1:500	ThermoFisher (A21208)

Figure S1

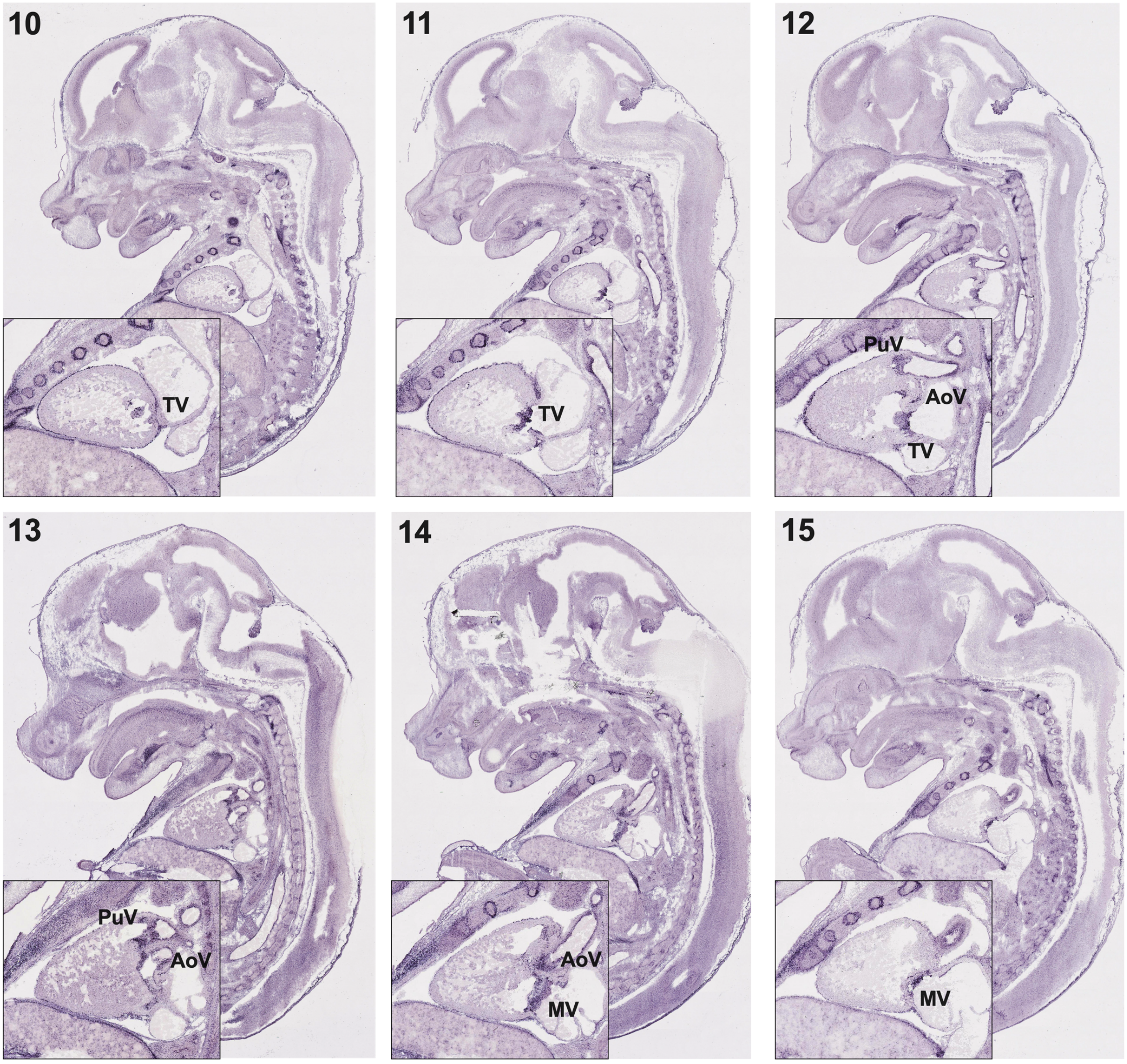

Figure S1: Expression profile of *Angptl2* RNA in Eurexpress.

In situ hybridizations on whole sagittal sections and their corresponding higher magnifications of the heart region (in the bottom left corner of the image) of wild-type mice embryos at E14.5 from the public database Eurexpress show a strong expression of *Angptl2* RNA in the four valves of the developing heart. Number in the top left corner of each image represents image number from Eurexpress: euxassay_007716. TV: tricuspid valve, PuV: pulmonary valve, AoV: aortic valve, MV: mitral valve.

Figure S2

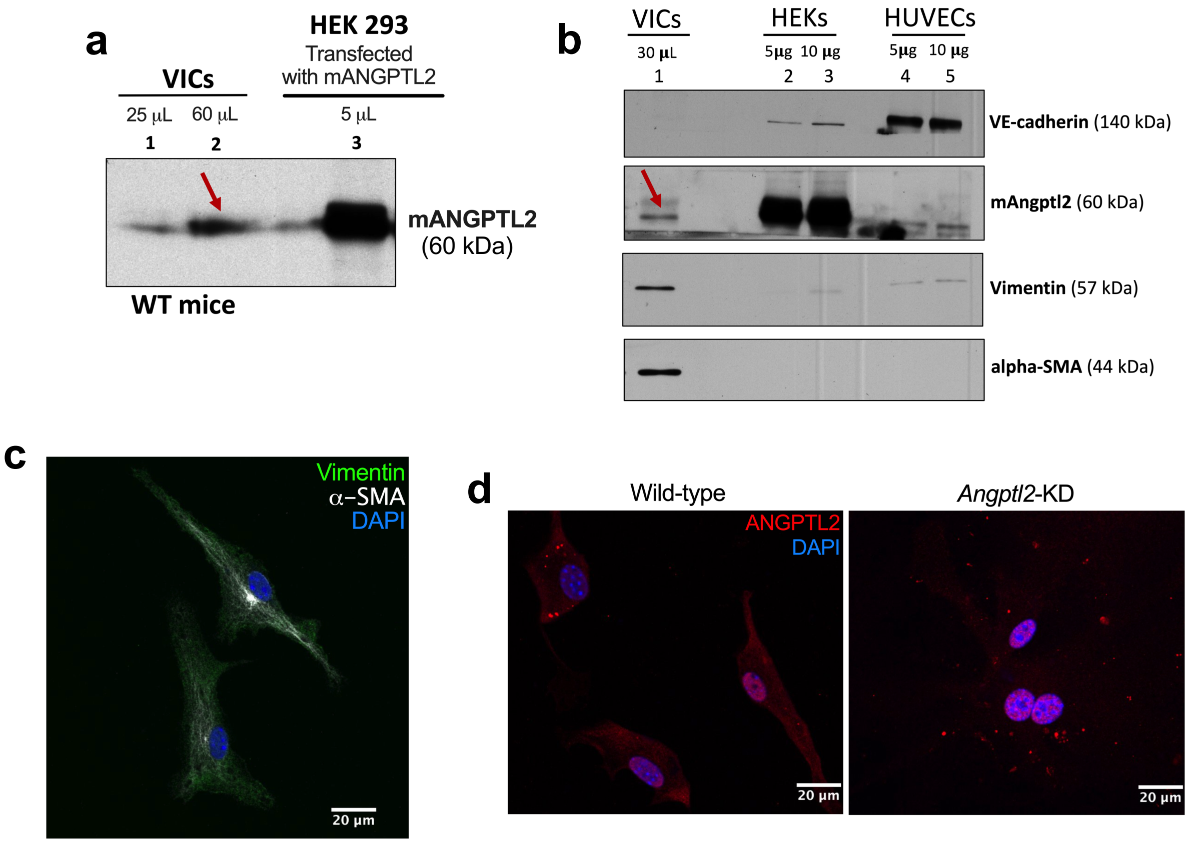

Figure S2: ANGPTL2 expression in sub-cultured mouse VICs.

(a) ANGPTL2 protein expression by Western Blot in VICs sub-cultured from AoV leaflets of WT mice (lines 1-2; indicated by a red arrow) compared to a positive control, *i.e.,* HEK293 cells transfected with a plasmid encoding the murine form of ANGPTL2 (line 3). **(b)** VE-cadherin, ANGPTL2, Vimentin and α-SMA expression by Western Blot in VICs from WT mice (line 1) compared to a positive control for Angptl2 expression, *i.e.*, HEK293 cells transfected with a plasmid encoding the murine form of Angptl2 (lines 2-3) and a positive control for endothelial markers, *i.e.*, HUVECs (lines 4-5). In addition to Angptl2, VICs expressed markers of (myo)fibroblasts such as vimentin and α-SMA (line 1). **(c)** Vimentin and α-SMA expression was also confirmed by immunofluorescence in VICs from WT mice. **(d)** Representative ANGPTL2 immunofluorescence in VICs from WT and Angptl2-KD mice. A cytoplasmic ANGPTL2 signal is only observed in WT VICs, but not in the negative control, *i.e.*, VICs from Angptl2-KD mice; Of note, the nuclear red-ANGPTL2 staining observed in cultured VICs from WT and Angptl2-KD mice was a non-specific signal. All images are representative of 3 independent experiments.

Figure S3

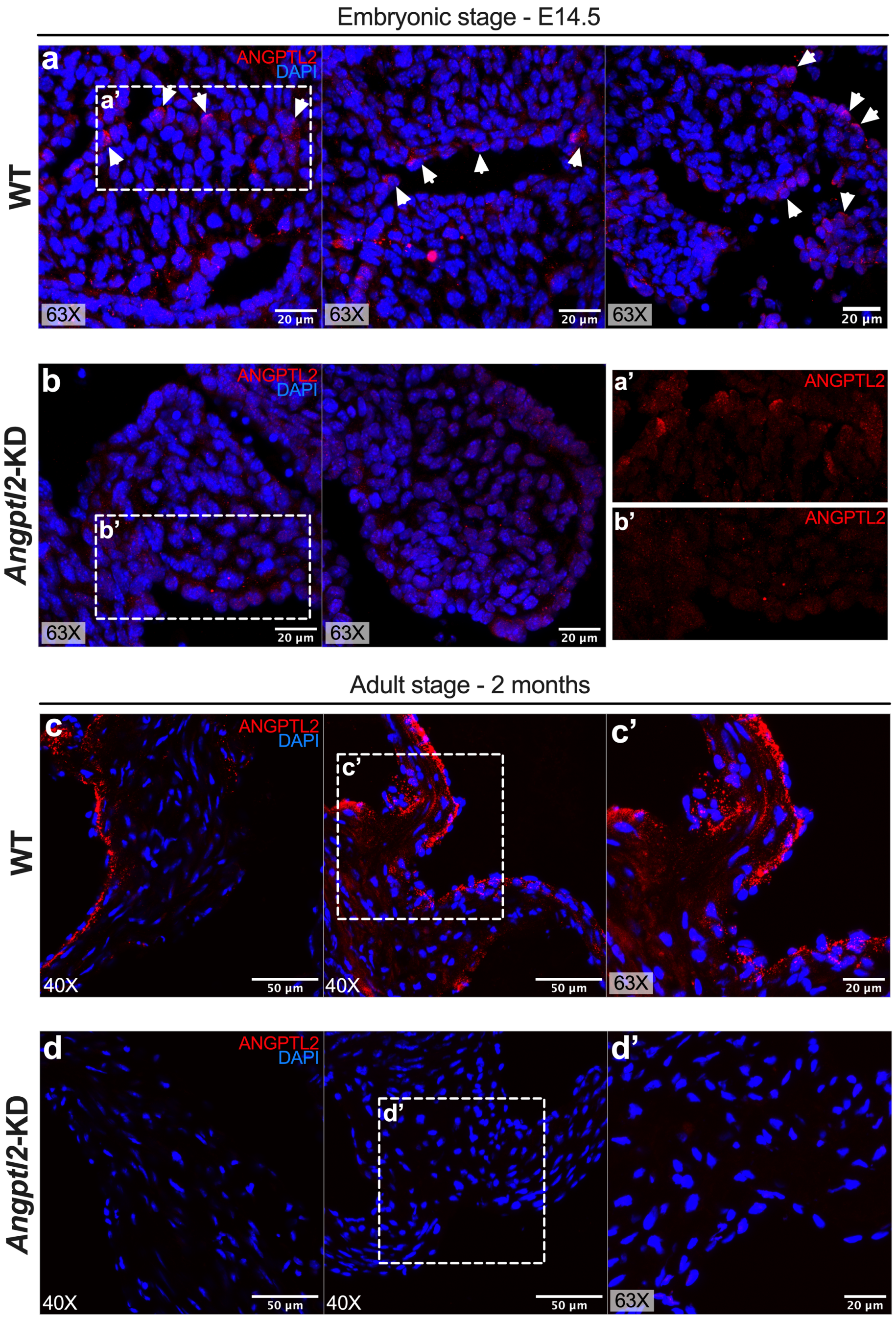

Figure S3: Specificity of ANGPTL2 staining of E14.5 and 2 month-old mice AoV.

(a) Representative ANGPTL2 protein expression by immunofluorescence in AoV sections at E14.5 from WT mice. White arrows show the specific expression of ANGPTL2 in the cytoplasm of valve cells at the border of the leaflet; Image for ANGPTL2 expression is shown without DAPI staining in **(a')**. **(b)** The specificity of ANGPTL2 antibody was tested on AoV sections from *Angptl2*-KD mice at E14.5, which provided a systematic, non-specific signal in the nucleus, which was noticeable depending on the experiments; Image for ANGPTL2 expression is shown without DAPI staining in **(b')**. **(c)** Representative ANGPTL2 protein expression by immunofluorescence in AoV sections from 2-month old WT mice; Higher magnification (X63) is shown in **(c')**. **(d)** Representative ANGPTL2 protein expression by immunofluorescence in AoV sections from 2-month old *Angptl2*-KD mice; Higher magnification (X63) is shown in **(d')**. All images are representative of 3 independent experiments.

Figure S4

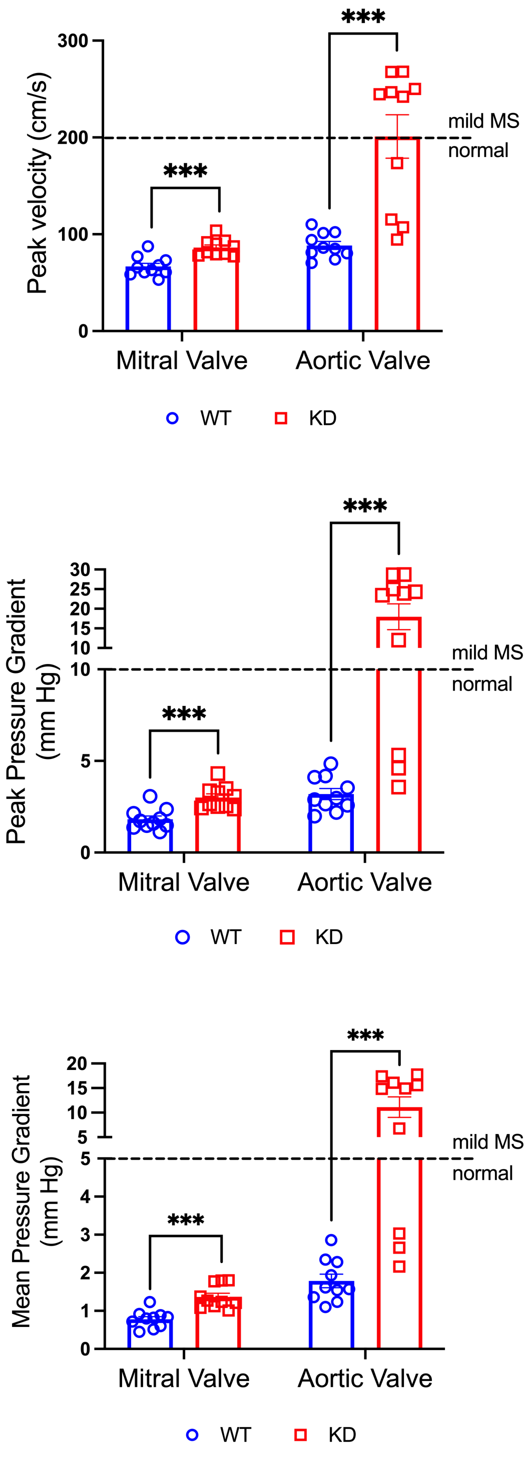

Figure S4: Evaluation of the mitral valve function by echo in *Angptl2*-KD mice.

Echocardiographic measurements of the peak velocity (Vmax), the peak and the mean pressure gradient of the mitral valve, and comparison with the parameters for aortic valve in male (n=5) and female (n=5) *Angptl2*-KD mice and WT littermates at 2 month-old (n=10 mice *per* genotype). Data are means±SEM of *n* mice. *: p<0.05 determined with Mann-Whitney U test.

Figure S5

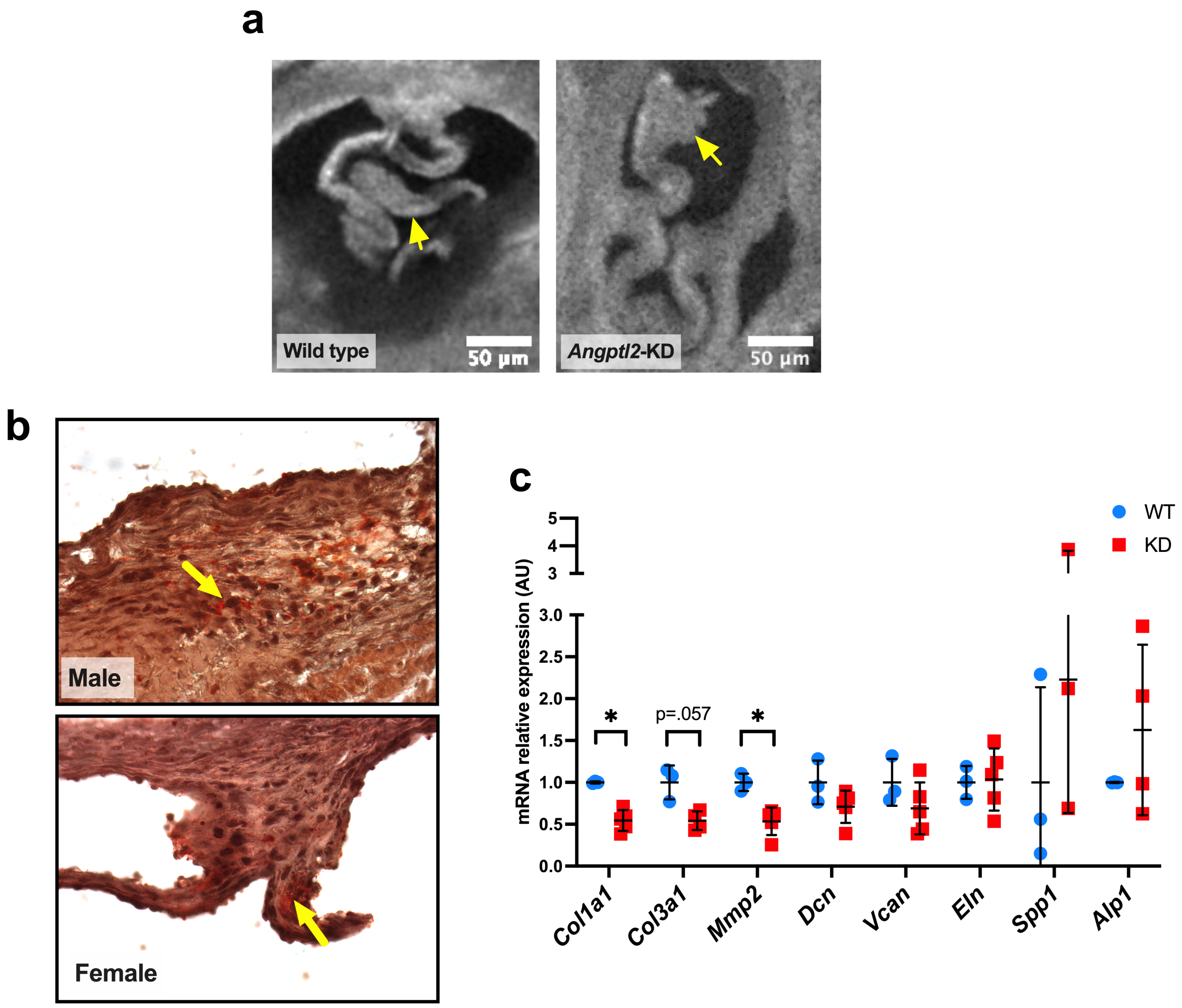

Figure S5 : AVS is still present in 7-10 month-old male Angptl2-KD mice.

(a) Optical coherence tomography (OCT) imaging of AoV from histological sections of 7-month old WT and *Angptl2*-KD mice. Leaflet thickening in *Angptl2*-KD mice compared with WT mice is indicated by yellow arrows. Images are representative of n=3 independent experiments. **(b)** Representative AoV sections (of n=3 independent experiments) from 7-month old male and female *Angptl2*-KD mice stained using alizarin red. Yellow arrows indicate calcium deposition (in red). **(c)** Gene expression by quantitative RT-qPCR in aortic valve leaflets from 10-month old male *Angptl2*-KD mice compared to their WT littermates, data are means±SEM of n=3 WT mice and n=3-5 *Angptl2*-KD mice. *: $p<0.05$ determined with Mann-Whitney U test.

Figure S6

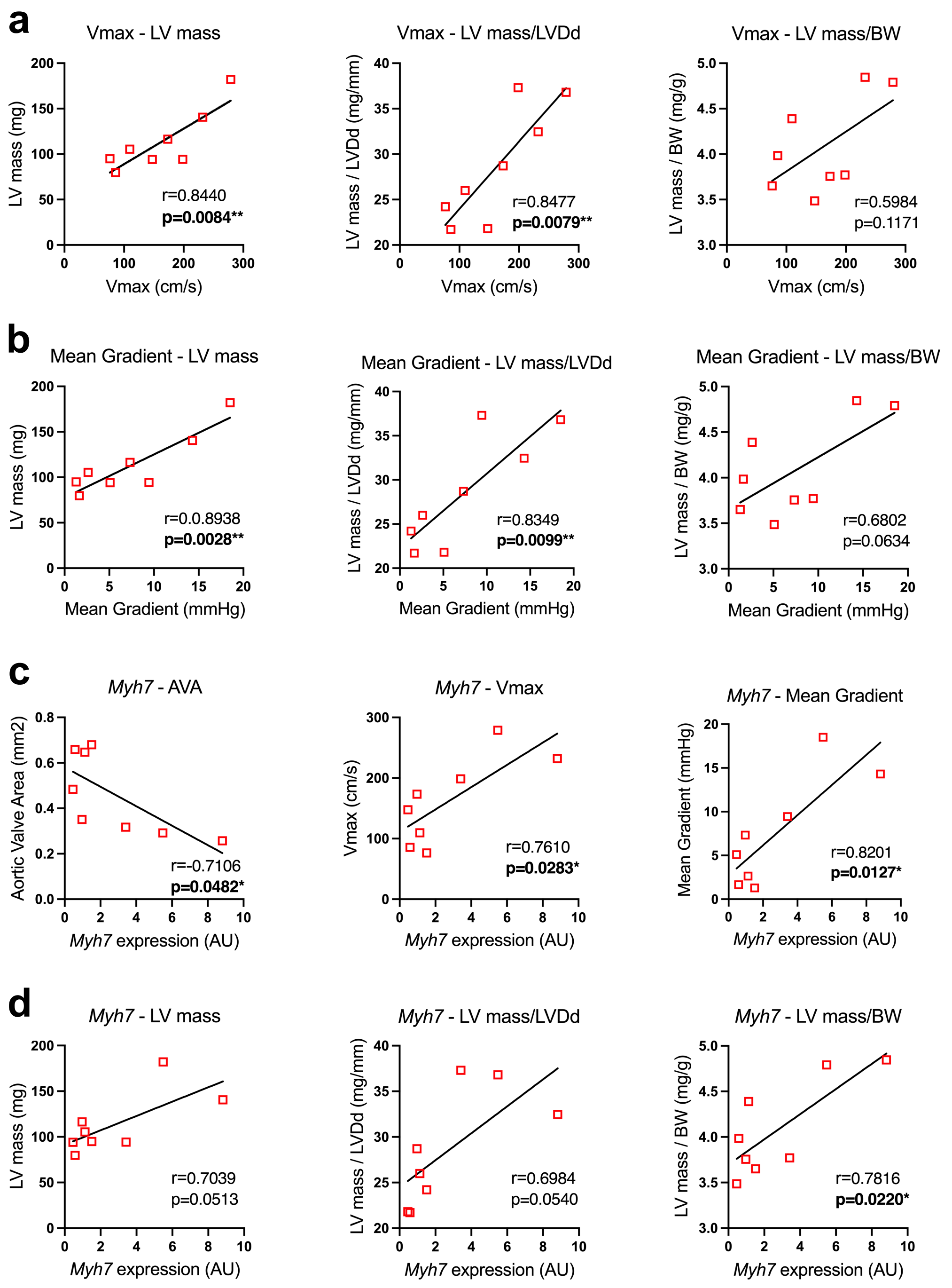

Figure S6: Correlation between the severity of AVS and cardiac hypertrophy in *Angptl2*-KD mice.

(a) Linear correlations between left ventricle (LV) mass, LV mass/LV dimension at end cardiac diastole (LVDd) ratio and LV mass/body weight (BW) ratio and the maximal velocity of AoV (Vmax). **(b)** Linear correlations between LV mass, LV mass/LVDd ratio and LV mass/BW ratio and the mean pressure gradient of the AoV. **(c)** Linear correlations between the AoV area (AVA), the Vmax and the mean gradient and *Myh7* mRNA levels in AoV leaflets. **(d)** Linear correlations between LV mass, LV mass/LVDd ratio and LV mass/BW ratio and *Myh7* mRNA levels in AoV leaflets. Data are means±SEM of n=8 mice (5 male and 3 female *Angptl2*-KD mice). *: $p<0.05$ determined with Spearman correlation tests.

Figure S7

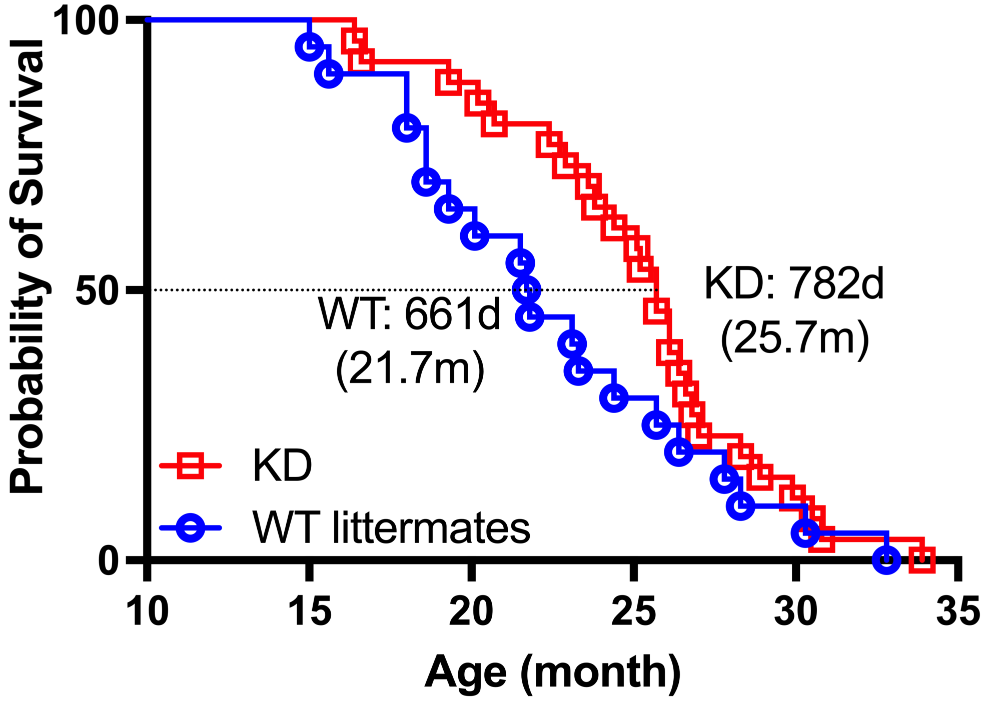

Figure S7: Survival curve of *Angptl2*-KD mice.

Evaluation of the lifespan of male *Angptl2*-KD mice (median survival age: 25.7 months; n=25) compared to WT littermates (median survival age: 21.7 months; n=19).

Figure S8

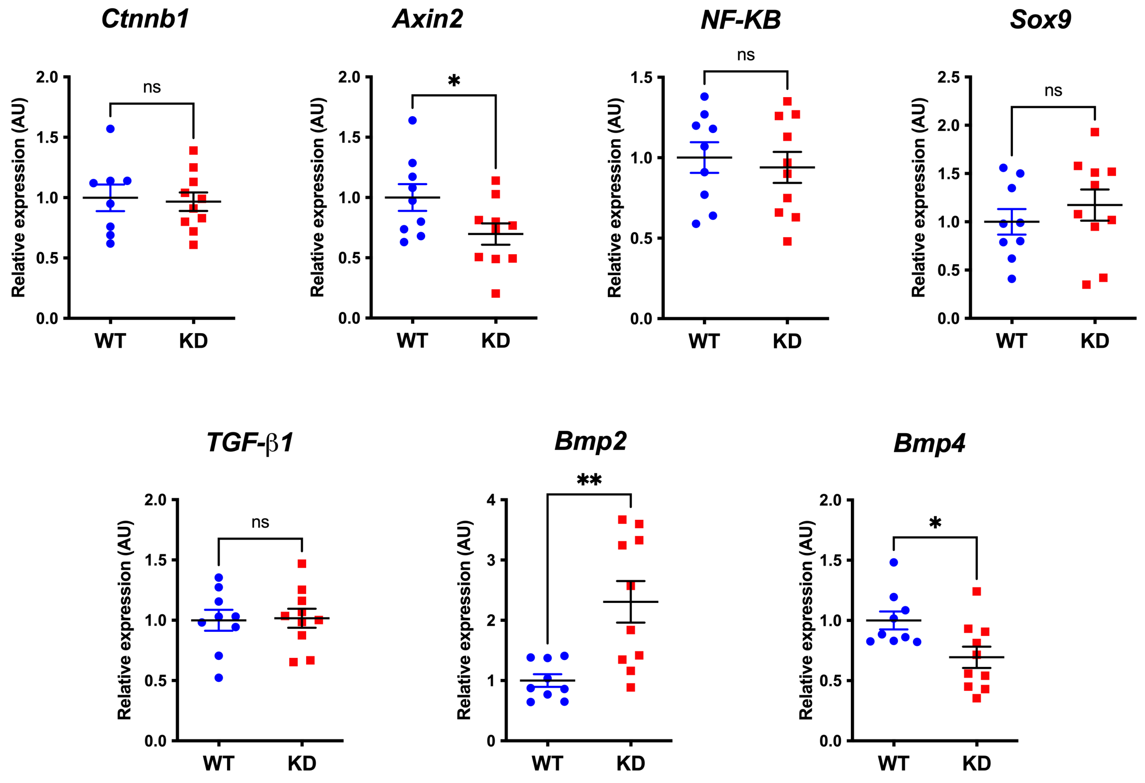

Figure S8: Changes in gene expression in AoV leaflets from *Angptl2*-KD mice.

Gene expression by quantitative RT-qPCR in aortic valve leaflets from 4-month old males and females *Angptl2*-KD compared to their WT littermates. Data are means±SEM of n=8-9 WT mice and n=10 *Angptl2*-KD mice. Males (n=4-5) and females (n=4-5) are pooled. *: p<0.05 determined with t-test or Mann-Whitney U test according to the normality of distribution.

Figure S9

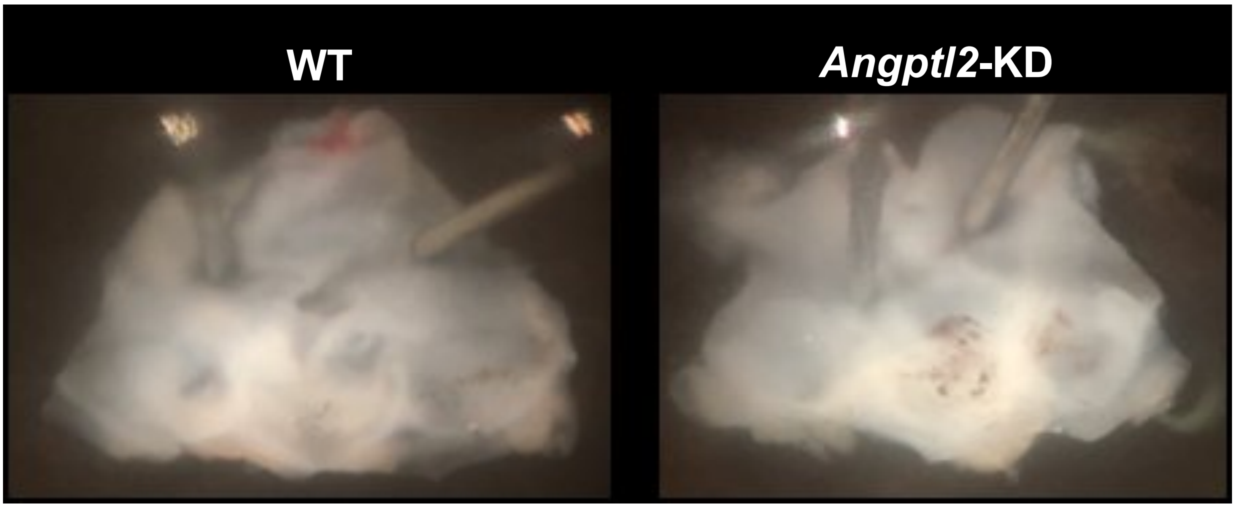

Figure S9: Increased black deposits observed in AoV leaflets from *Angptl2*-KD mice.

Representative images (of n=3 independent experiments) of whole mount preparations of AoV leaflets from 2-month old *Angptl2*-KD mice show increased black deposits – that could be melanin, as reported in the literature in *Axin2*-KO mice – compared to age-matched WT mice.

Figure S10

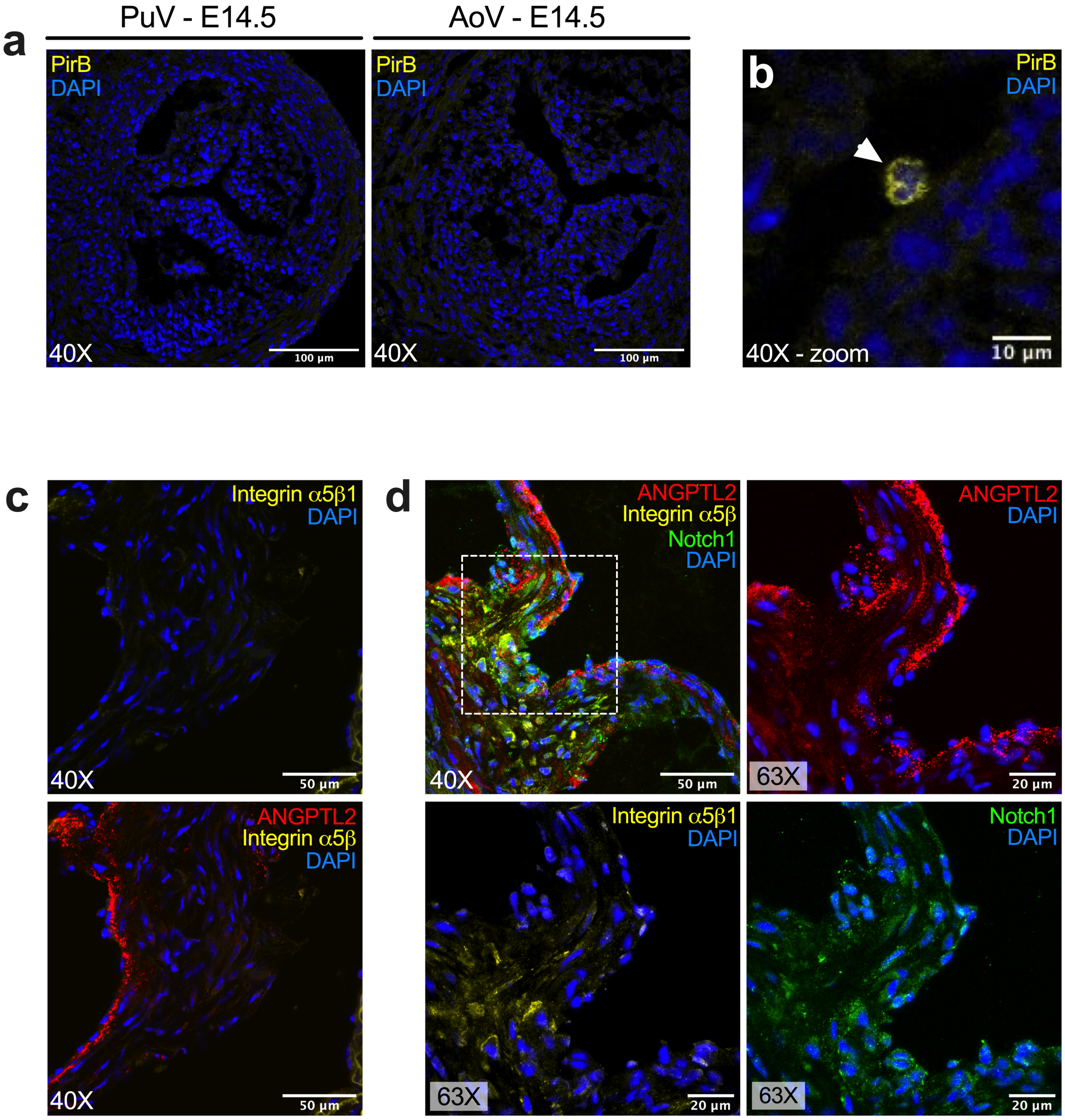

Figure S10: PirB and Integrin α5β1 expression in embryonic and adult WT mice AoV.

(a) Representative PirB protein expression by immunofluorescence in PuV and AoV sections from E14.5 WT embryo; PirB is not detected in valve leaflets at that age. **(b)** PirB was only detected in some polylobed nucleus, characteristics of neutrophils, scattered across the valve leaflets (indicated with a white arrowhead). **(c)** Representative Integrin α5β1 and ANGPTL2 protein expression by immunofluorescence in AoV leaflet from 2-month old WT mice. **(d)** Representative Integrin α5β1, ANGPTL2 and Notch1 protein expression by immunofluorescence in AoV pillar from 2-month old WT mice. All images are representative of 3 independent experiments.

Figure S11

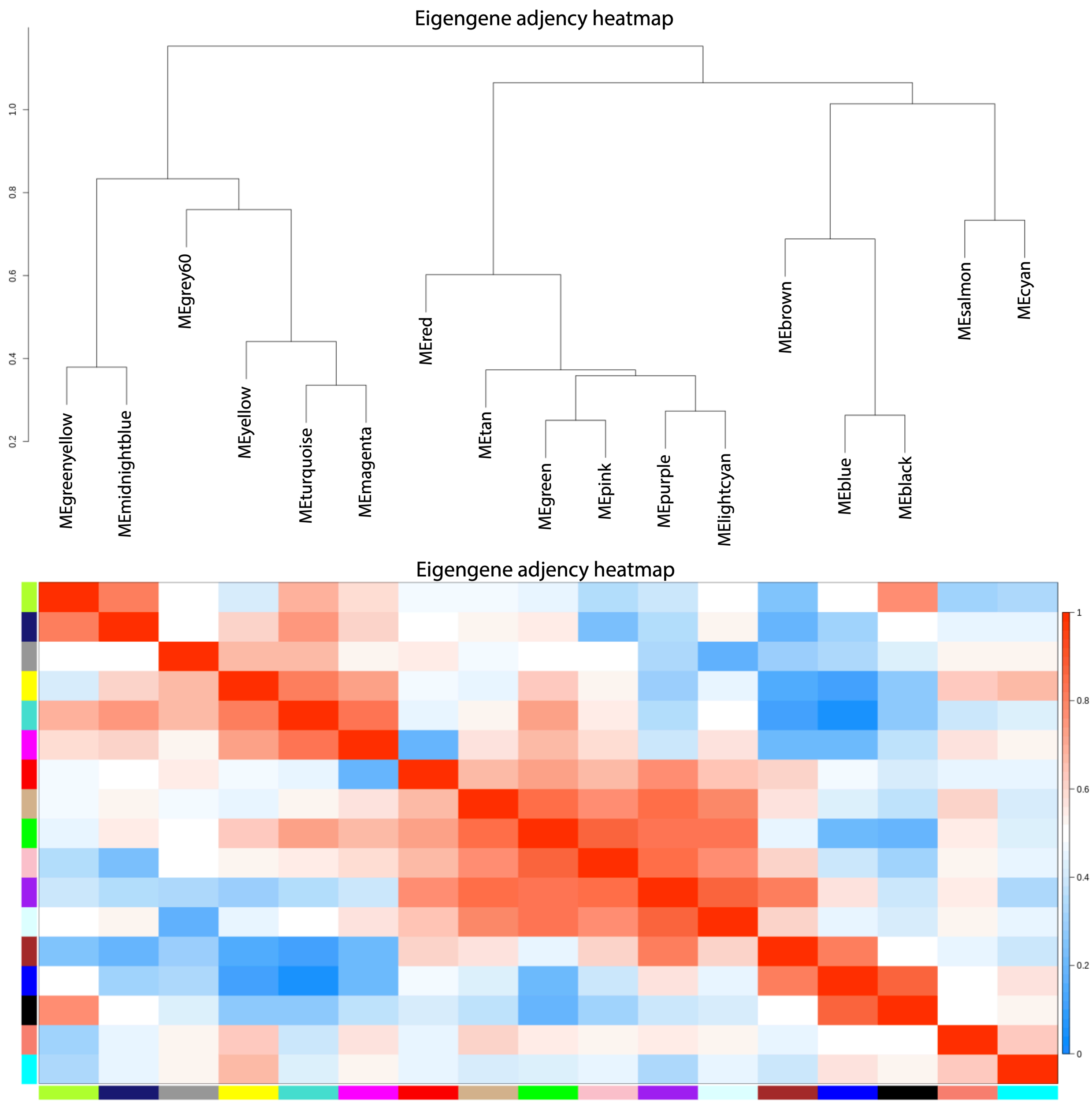

Figure S11: WGCNA analysis identified 17 heart valve genetic modules.

Figure S12

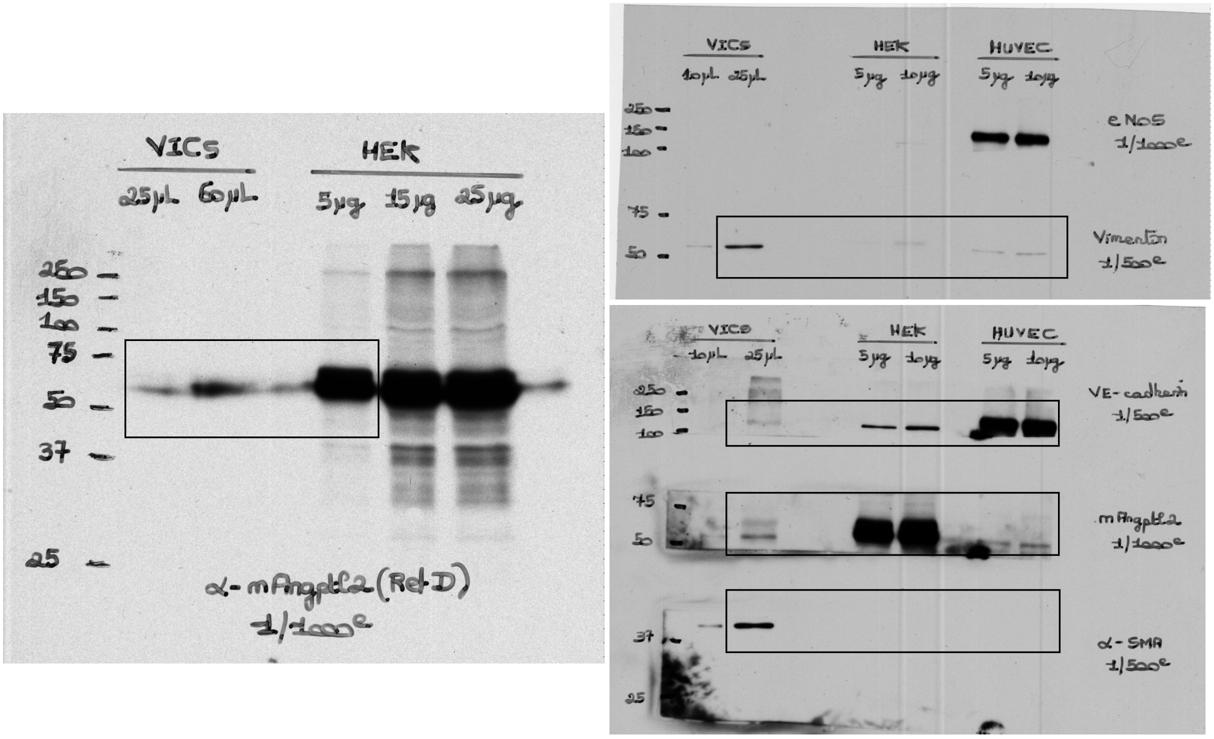

Figure S12: Unedited blots/gels for Figure S2.

Supplementary Methods

OCT Imaging Each valve was embedded in 4% agarose cylindrical blocks for serial imaging. An in-house automated high-throughput histology setup combined with a swept-source optical coherence tomography (SS- OCT) microscope was used to image the agarose embedded valves. The SS-OCT system was reported previously (1). The swept-source laser was operated at a central wavelength of 1310 nm with a tuning bandwidth of100 nm (Axsun, 1310 Swept Source Engine). A cropped Blackman apodization function was used to reduce the side lobes caused by the rectangular shape of the swept-source spectrum. A 3× microscope objective (Thorlabs, LSM04 Scan Lens) was enclosed in a 3-D printed watertight immersion chamber ending with a glass coverslip to protect the scanning lens from water. The system resolutions in water were 8 μm laterally and 10 μm axially. Its sensitivity was 98.5 dB, and its sensitivity roll-off along depth was −0.01 dB⁄μm.

Images were acquired sequentially along x/y by moving a XY stage. At each motor position, the sampling beam was raster scanned over the objective's field of view using galvanometric mirrors. This resulted in a mosaic of volumetric OCT tiles. The volumes were assembled, and a maximum intensity projection was done to recover the final images.

Supplementary Reference

1. Lefebvre J, et al. Whole mouse brain imaging using optical coherence tomography: reconstruction, normalization, segmentation, and comparison with diffusion MRI. *Neurophotonics*. 2017;4(4):041501-041501.
