# Supplementary material for: Angiopoietin-like 2 is essential to aortic valve development in mice
Source: Commun Biol. 2022 Nov 21;5:1277. doi: 10.1038/s42003-022-04243-6 (PMC9681843; doi:10.1038/s42003-022-04243-6)
Supplement: Supplementary file 3 — Description of Additional Supplementary Files [file 42003_2022_4243_MOESM3_ESM.pdf]

Description of Additional Supplementary Files

File name: Supplementary Data 1-4
Description:

Supplementary Data 1: Genes significantly regulated in human adult vs. Fetal aortic valves.

Supplementary Data 2: WGCNA turquoise gene module.

Supplementary Data 3: Pathway enrichment of the WGCNA turquoise gene module.

Supplementary Data 4: Source data for main Figures.
