# Supplementary material for: Angiopoietin-like 2 is essential to aortic valve development in mice
Source: Commun Biol. 2022 Nov 21;5:1277. doi: 10.1038/s42003-022-04243-6 (PMC9681843; doi:10.1038/s42003-022-04243-6)
Supplement: Supplementary file 5 — Reporting Summary [file 42003_2022_4243_MOESM5_ESM.pdf]

nature portfolio

Corresponding author(s): Pauline LABBÉ

Last updated by author(s): Nov 8, 2022

Reporting Summary

Nature Portfolio wishes to improve the reproducibility of the work that we publish. This form provides structure for consistency and transparency in reporting. For further information on Nature Portfolio policies, see our Editorial Policies and the Editorial Policy Checklist.

Statistics

For all statistical analyses, confirm that the following items are present in the figure legend, table legend, main text, or Methods section.

n/a	Confirmed	
☐	☒	The exact sample size (n) for each experimental group/condition, given as a discrete number and unit of measurement
☐	☒	A statement on whether measurements were taken from distinct samples or whether the same sample was measured repeatedly
☐	☒	The statistical test(s) used AND whether they are one- or two-sided<br>*Only common tests should be described solely by name; describe more complex techniques in the Methods section.*
☒	☐	A description of all covariates tested
☐	☒	A description of any assumptions or corrections, such as tests of normality and adjustment for multiple comparisons
☐	☒	A full description of the statistical parameters including central tendency (e.g. means) or other basic estimates (e.g. regression coefficient) AND variation (e.g. standard deviation) or associated estimates of uncertainty (e.g. confidence intervals)
☐	☒	For null hypothesis testing, the test statistic (e.g. F, t, r) with confidence intervals, effect sizes, degrees of freedom and P value noted<br>*Give P values as exact values whenever suitable.*
☒	☐	For Bayesian analysis, information on the choice of priors and Markov chain Monte Carlo settings
☒	☐	For hierarchical and complex designs, identification of the appropriate level for tests and full reporting of outcomes
☐	☒	Estimates of effect sizes (e.g. Cohen's d, Pearson's r), indicating how they were calculated

Our web collection on statistics for biologists contains articles on many of the points above.

Software and code

Policy information about availability of computer code

Data collection	StepOnePlus™ Software v2.3 (qPCR); GE Vivid 7 Dimension (echo); ZEN 2012 Zeiss (confocal)
Data analysis	GraphPad Prism 9 for macOS; ImageJ version 2.0; Microsoft Excel for Mac version 16.53; Microsoft PowerPoint for Mac version 16.63.1

For manuscripts utilizing custom algorithms or software that are central to the research but not yet described in published literature, software must be made available to editors and reviewers. We strongly encourage code deposition in a community repository (e.g. GitHub). See the Nature Portfolio guidelines for submitting code & software for further information.

Data

Policy information about availability of data

All manuscripts must include a data availability statement. This statement should provide the following information, where applicable:

- Accession codes, unique identifiers, or web links for publicly available datasets
- A description of any restrictions on data availability
- For clinical datasets or third party data, please ensure that the statement adheres to our policy

Gene expression profiling by Illumina array that support the findings of this study was downloaded from the Gene Expression Omnibus under accession number GSE45821. In situ hybridizations on whole sagittal sections of wild-type mice embryos at E14.5 that support the findings of this study was downloaded from the public database Eurexpress under accession number euxassay_007716. The pBlueScript SK (+) vector used in this study is available from Addgene under ID 212205. Source data are available in the Supplementary Data 1-4. Unedited blots/gels for Figure S2 are available in the Figure S12.
Data supporting the findings of this study are available within the paper and its supplementary information files, or are available from the corresponding author on reasonable request.

Field-specific reporting

Please select the one below that is the best fit for your research. If you are not sure, read the appropriate sections before making your selection.

☒ Life sciences ☐ Behavioural & social sciences ☐ Ecological, evolutionary & environmental sciences

For a reference copy of the document with all sections, see nature.com/documents/nr-reporting-summary-flat.pdf

Life sciences study design

All studies must disclose on these points even when the disclosure is negative.

Sample size	n=5 was the minimal amount of samples to account for statistical significance, except when constrained by the availability of animal subjects.
Data exclusions	No data were excluded.
Replication	The experimental findings showing only one image (Figure 1, in situ hybridization experiments; Angptl2 expression in valves, etc.) were replicated at least three times. Otherwise, replicates of experiments described in this paper are shown or included in statistics.
Randomization	N/A
Blinding	The investigators were blinded to group allocation during analysis, in order to remove as much experimenter biases as possible.

Reporting for specific materials, systems and methods

We require information from authors about some types of materials, experimental systems and methods used in many studies. Here, indicate whether each material, system or method listed is relevant to your study. If you are not sure if a list item applies to your research, read the appropriate section before selecting a response.

Materials & experimental systems

n/a	Involved in the study	
☐	☒	Antibodies
☐	☒	Eukaryotic cell lines
☒	☐	Palaeontology and archaeology
☐	☒	Animals and other organisms
☒	☐	Human research participants
☒	☐	Clinical data
☒	☐	Dual use research of concern

Methods

n/a	Involved in the study	
☒	☐	ChIP-seq
☒	☐	Flow cytometry
☒	☐	MRI-based neuroimaging

Antibodies

Antibodies used	mAngptl2 (AF1444, R&D Systems); CD31 (ab28364, Abcam); CD31 (AF3628, R&D Systems); p21 (ab188224, Abcam); Ki67 (ab15580, Abcam); Vimentin (VP-V683, Vector Laboratories); a-SMA (A5228, Sigma-Aldrich); activated Notch1 (ab8925, Abcam); Notch1 (ab52627, Abcam); Integrin a5b1 (MAB2514, Sigma-Aldrich); PirB (ab284407, Abcam); Alexa fluor-647 anti-rabbit (#A31573, ThermoFisher); Alexa fluor-488 anti-rat (#A21208, ThermoFisher); Alexa fluor-555 anti-mouse (#A31570, ThermoFisher); Alexa fluor-555 anti-goat (#A21432, ThermoFisher).
Validation	mAngptl2 (AF1444, R&D Systems) has been referenced in publications, and specificity was validated using Angptl2-KD tissues. Other antibodies have been referenced in publications using mice tissues (ab28364; VP-V683; ab8925) and/or validated in mice tissues/or KO-validated by the manufacturer; information available on their website (AF3628; ab188224; ab15580; A5228; ab52627). In addition, negative controls were performed for every immunofluorescence experiments (same samples with secondary antibody without primary antibody).

Eukaryotic cell lines

Policy information about cell lines

Cell line source(s)	Valve interstitial cells (VICs) were isolated from WT and Angptl2-KD mice. HEK 293 and HUVECs cells were provided by ATCC.
Authentication	VICs and cell lines were used to test Angptl2 expression, and authenticated with specific markers.
Mycoplasma contamination	VICs were isolated from WT and Angptl2-KD mice, and tested by careful examination of the nucleus integrity by DAPI-stained cells on coverslips. HEK 293 and HUVECs cells were used as expected positive controls for Angptl2 and VE-cadherin expressions, respectively, in Supplemental Figure 2.

Commonly misidentified lines (See ICLAC register)	N/A

Animals and other organisms

Policy information about studies involving animals; ARRIVE guidelines recommended for reporting animal research

Laboratory animals	Male and female Angptl2-KD mice from our colony at ages between E9.5 and 10 months were used.
Wild animals	N/A
Field-collected samples	N/A
Ethics oversight	All animal experiments were performed in accordance with the "Guide for the Care and Use of Experimental Animals of the Canadian Council on Animal Care" and were approved by the Montreal Heart Institute Animal Research Ethics Committee (ET 2017-62-03; ET 2019-62-02).

Note that full information on the approval of the study protocol must also be provided in the manuscript.
